# Fast and Furious: Energetic Tradeoffs and Scaling of High-Speed Foraging in Rorqual Whales

**DOI:** 10.1093/iob/obac038

**Published:** 2022-08-27

**Authors:** William T Gough, David E Cade, Max F Czapanskiy, Jean Potvin, Frank E Fish, Shirel R Kahane-Rapport, Matthew S Savoca, K C Bierlich, David W Johnston, Ari S Friedlaender, Andy Szabo, Lars Bejder, Jeremy A Goldbogen

**Affiliations:** Hopkins Marine Station, Stanford University, Pacific Grove, CA 94305, USA; Hopkins Marine Station, Stanford University, Pacific Grove, CA 94305, USA; Hopkins Marine Station, Stanford University, Pacific Grove, CA 94305, USA; Saint Louis University, Saint Louis, MO 63103, USA; West Chester University, West Chester, PA 19383, USA; California State University, Fullerton, Fullerton, CA 90032, USA; Hopkins Marine Station, Stanford University, Pacific Grove, CA 94305, USA; Oregon State University, Corvallis, OR 97331, USA; Duke University, Durham, NC 27708, USA; University of California, Santa Cruz, Santa Cruz, CA 94720, USA; Alaska Whale Foundation, Sitka, AK, 99835, USA; Hawaii Institute of Marine Biology, University of Hawaii at Manoa, Kaheohe, HI 96822, USA; Department of Bioscience, Aarhus University, Aarhus 8000, Denmark; Hopkins Marine Station, Stanford University, Pacific Grove, CA 94305, USA

## Abstract

Although gigantic body size and obligate filter feeding mechanisms have evolved in multiple vertebrate lineages (mammals and fishes), intermittent ram (lunge) filter feeding is unique to a specific family of baleen whales: rorquals. Lunge feeding is a high cost, high benefit feeding mechanism that requires the integration of unsteady locomotion (i.e., accelerations and maneuvers); the impact of scale on the biomechanics and energetics of this foraging mode continues to be the subject of intense study. The goal of our investigation was to use a combination of multi-sensor tags paired with UAS footage to determine the impact of morphometrics such as body size on kinematic lunging parameters such as fluking timing, maximum lunging speed, and deceleration during the engulfment period for a range of species from minke to blue whales. Our results show that, in the case of krill-feeding lunges and regardless of size, animals exhibit a skewed gradient between powered and fully unpowered engulfment, with fluking generally ending at the point of both the maximum lunging speed and mouth opening. In all cases, the small amounts of propulsive thrust generated by the tail were unable to overcome the high drag forces experienced during engulfment. Assuming this thrust to be minimal, we predicted the minimum speed of lunging across scale. To minimize the energetic cost of lunge feeding, hydrodynamic theory predicts slower lunge feeding speeds regardless of body size, with a lower boundary set by the ability of the prey to avoid capture. We used empirical data to test this theory and instead found that maximum foraging speeds remain constant and high (∼4 m s^–1^) across body size, even as higher speeds result in lower foraging efficiency. Regardless, we found an increasing relationship between body size and this foraging efficiency, estimated as the ratio of energetic gain from prey to energetic cost. This trend held across timescales ranging from a single lunge to a single day and suggests that larger whales are capturing more prey—and more energy—at a lower cost.

## Introduction

Energy is a key currency for all animal life. The efficient acquisition and use of energy strongly influences the fitness of individuals (Boyd and Hoelzel, 2002; Christiansen et al., 2014; Crossin et al., 2014; Chimienti et al., 2020). Essential behaviors and functions incur energetic costs that must be balanced by energy gain. Excess energy usage relative to energy gain yields deficits that draw down energy reserves (i.e., lipid stores), and in extreme cases may result in physiological compensation such as immune system depression (Martin et al., 2008) or cessation of reproduction or migration (Svedäng and Wickström, 1997). In contrast, an energetic surplus beyond the basic costs of life can provide increased capacity to carry out essential functions, adapt to changing environmental conditions, and increase reproductive fitness in a variety of ways including higher fecundity and enhanced provisioning of young (Sebens, 1982; Priede, 1985; Sokolova et al., 2012).

Animals must balance foraging strategies that minimize energetic costs while maximizing energy intake (Pyke et al., 1977; Hazen et al., 2015). Predators that rely on active prey chasing and capture can increase the energetic efficiency of foraging (*FE* = energy in/energy out) by decreasing the cost of locomotion and/or increasing the energetic yield from prey acquisition. For animals that typically capture one prey item at a time (particulate feeders), increased energy yield can be achieved by capturing larger or more numerous prey (Kerr, 1971; Werner and Hall, 1974; Goldbogen et al., 2019b) as, for example, facilitated by echo-location (Goldbogen and Madsen, 2018). On the other hand, suspension feeding animals, which sieve or use cross-flow filtration to remove relatively small and numerous prey from water flows, provide useful study systems to explore mechanisms that determine energy balance (Sebens, 1982). Sessile suspension feeders may exhibit low-cost energetics because there are no locomotor costs, but energy yield is limited to prey abundance and distributions in proximate flows (Okamura, 1990).

**Fig. 1:**
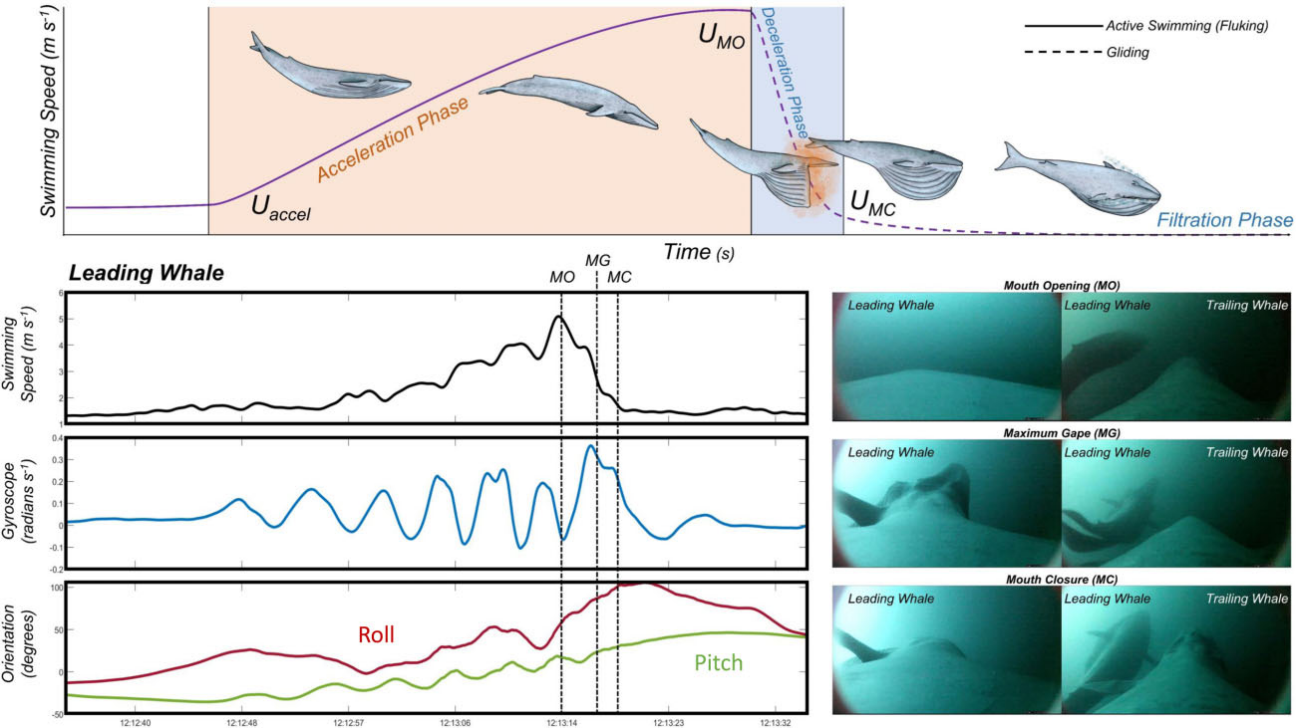
Top shows a schematic overview of a foraging lunge swimming speed trace. The light orange and blue areas correspond to the acceleration (*ΔT_accel_*) and deceleration (*ΔT_decel_*) phases of the lunge, respectively. This lunge does not display an adjustment phase (*ΔT_adjust_*). Bottom shows kinematic data and corresponding camera views for paired blue whales lunge feeding on krill. The images on the right are taken from CATS biologging tags deployed on a pair of blue whales, with the data traces corresponding to the leading animal in the pair. Each set of images from top to bottom correspond to specific times during the lunge and are represented in the data traces as dotted lines. These times are (1) the point of mouth opening at the beginning of the lunge (MO), (2) the maximum gape during the lunge (MG), and (3) the mouth closure at the end of the lunge (MC).

**Fig. 2:**
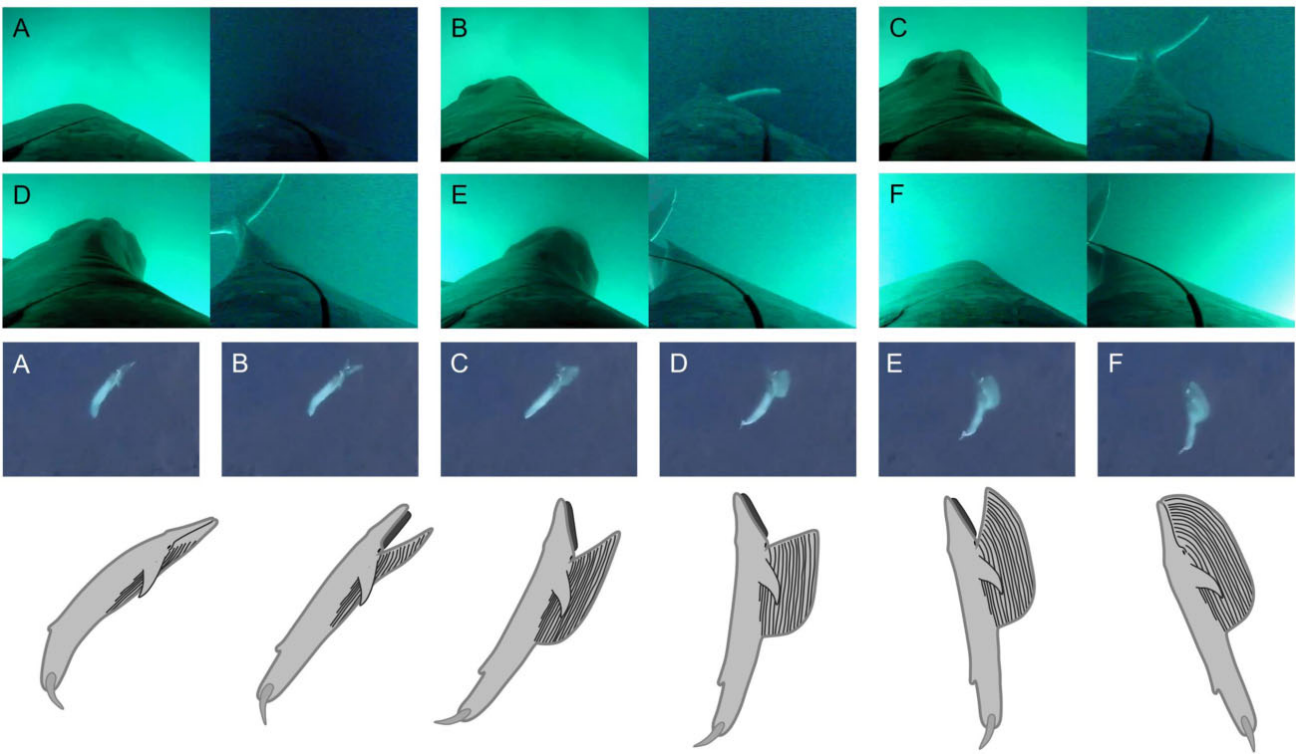
Representative views of rorqual lunges from tag and UAS camera views and interpretative schematic of body kinematics. The stages of the feeding lunge follow the same general pattern stepwise from (A) tail in a bottom-of-beat position with the mouth closed and the body arched downward, (B) tail returns to a neutral position and the head begins to rise as the mouth opens, (C) The tail moves to a top-of-beat position as the mouth opens further, (D) the tail remains in a top-of-beat position as the mouth reaches maximum gape, (E) the tail begins to move toward a more neutral position as the mouth begins to close, (F) the tail reaches a neutral or slightly elevated position as the mouth closes.

In contrast, ram filter feeders (RFF) require forward locomotion to drive prey-laden water through large filtration apparatuses and consequently experience high energetic costs due to high drag (Werth, 2004; Potvin et al., 2021). However, if RFF can find sufficiently dense prey patches, energy intake can exceed energy costs by up to several orders of magnitude in the most efficient foragers (Hazen et al., 2015; Goldbogen et al., 2019b). The high drag required for ram filter feeding forces most aquatic animals to forage at slow, steady speeds and keep energy costs down (Sims, 2000; Werth, 2004; Simon et al., 2009; Motta et al., 2010). This kind of continuous RFF has evolved independently in many marine vertebrate lineages including cartilaginous fishes, bony fishes, and balaenid whales (Friedman, 2012). Continuous RFF first evolved in multiple fish lineages as early as the Jurassic Period. In contrast, the rorqual whales (Balaenopteridae), evolved recently (<5 mya) and rapidly achieved the largest body sizes of all time (Slater et al., 2017). Their unique RFF strategy, termed lunge feeding, is much more intermittent and dynamic (Figs. 1 and 2) than continuous RFF (Goldbogen et al., 2017a) in a majority of circumstances. Understanding the precise biomechanics and energetics of lunge feeding is critical to bridge the gap between evolution, ecology, and physiology and will elucidate the rorqual's unique path to marine gigantism (Alexander, 1998; Gearty et al., 2018; Goldbogen et al., 2019b).

Rorqual lunge feeding involves accelerating towards a prey patch, opening the mouth at high speed to engulf a large volume of prey-laden water, then filtering out the water and swallowing the bolus of prey (Simon et al., 2012; Cade et al., 2016; Goldbogen et al., 2017a; Cade et al., 2020; Potvin et al., 2021). During the lunge, the ventral groove blubber (VGB) in the buccal cavity expands outward like a parachute (Shadwick et al., 2013), substantially increasing the drag on the animal's body and slowing it down while increasing the amount of water that can be engulfed (Potvin et al., 2021). Goldbogen et al. (2019b) demonstrated that even though lunge feeding involves high locomotor costs due to increased drag, the energetic gains for rorquals from increased engulfment capacity will greatly exceed the locomotor costs when dense prey patches are found. Because engulfment capacity has been shown to exhibit positive allometry both within and among rorqual species (Goldbogen et al., 2010; Kahane‐Rapport and Goldbogen, 2018; Kahane-Rapport et al., 2020), the energetic efficiency of rorqual foraging was predicted to increase with body size (Goldbogen et al., 2019b). However, this prediction lacked morphometric measurements for the tagged whales discussed in the study, relying instead on species averages. Thus, the scaling of lunge feeding kinematics and energetics, as directly correlated to body size, remains poorly understood within rorqual species that vary in size.

The incorporation of morphological and tag-derived kinematic data into computational physics-based models enables a first approximation of lunge feeding mechanics and energetics (Potvin et al., 2020; Potvin et al., 2021). The first models of lunge feeding were developed based on low sample-rate accelerometer data, which indicated that fin whales (*Balaenoptera physalus*) fluke continuously throughout the lunge while their mouth is open (Goldbogen et al., 2006). This “fluke-through” model suggested that fluking occurs during both the accelerative and decelerative portions of the lunge (Simon et al., 2012). Such accelerative-decelerative fluking has been shown in fish-feeding lunges, but rarely in krill-feeding lunges (Cade et al., 2016; Cade et al., 2020). An alternative to this “fluke-through” scenario has been proposed for krill lunges in which body inertia plays a more dominant role, whereby an animal accelerates up to lunging speeds on approach to the prey and begins to coast as it opens its mouth (Potvin et al., 2020; Potvin et al., 2021). From an optimal energetics perspective, lunging in this “acceleration-coasting” fashion provides the greatest benefit for the lowest cost, as active swimming against the increased drag of the open mouth would incur additional cost (Cade et al, 2020; Potvin et al., 2020).

In this study, we hoped to: (1) combine simultaneous tag-based kinematic and video data to determine whether whales lunge feeding on krill employ “fluke-through” engulfment, “acceleration-coasting” engulfment, or some combination of the two, (2) expand on the kinematic measurements performed by Cade et al. (2016) with a greater number of individuals and species and increased granularity for variables such as swimming speed, and (3) broaden the scale-dependent energetic models estimated from Goldbogen et al. (2019b) to multiple time-scales with expanded morphometrics (e.g., body length, jaw area) obtained from unoccupied aerial systems (UAS; i.e., drones). Our results provide a more complete understanding of this unique foraging strategy and the link between extreme body size and foraging efficiency.

## Methods

### Study species, location, and animal-borne tags

We deployed multi-sensor tags on krill-feeding individuals from several rorqual species: Antarctic minke whales (*Balaenoptera bonaerensis*) off the western Antarctic Peninsula, humpback whales (*Megaptera novaeangliae*) off the western Antarctic Peninsula and the coast of Monterey, CA, a fin whale in Monterey Bay, and blue whales (*Balaenoptera musculus*) in Monterey Bay and Southern California Bight (Goldbogen et al., 2019b; Cade et al., 2021a). We focused our analyses on krill-feeding individuals to maintain kinematic consistency between species and individuals. All work was performed under federal permits and in accordance with university IACUC procedures (See the *Acknowledgements* section below). Built by Customized Animal Tracking Solutions (CATS), the tags include the following sensors and sampling rates: accelerometers (400 Hz), gyroscopes (50 Hz), magnetometers (50 Hz), and pressure (10 Hz). All tags included either a single forward-facing or a single forward- and a single rear-facing camera. All data was decimated down to 10 Hz and we corrected for whale body orientation using custom-written scripts in Matlab 2014a and 2020 (Cade et al., 2021b). We determined swimming speed using a regression between the orientation-corrected depth rate during high pitch-angle swimming segments (OCDR = vertical velocity from the pressure sensor divided by the sine of the body pitch angle from inertial sensors) (Miller et al., 2004) and the amplitude of tag vibrations (Cade et al., 2018). More information on tag deployment methods and the type of tag used here can be found in Goldbogen et al. (2017b).

### Morphological measurements

We used UAS to take nadir images of tagged whales at known altitudes using the methods outlined by Bierlich et al. (2021). For each animal, images were selected where the lower jaw, fluke notch, and sides were clearly visible at or very near the water's surface. We used these images for morphological analysis in the software package MorphoMetriX (Torres and Bierlich, 2020).

**Fig. 3:**
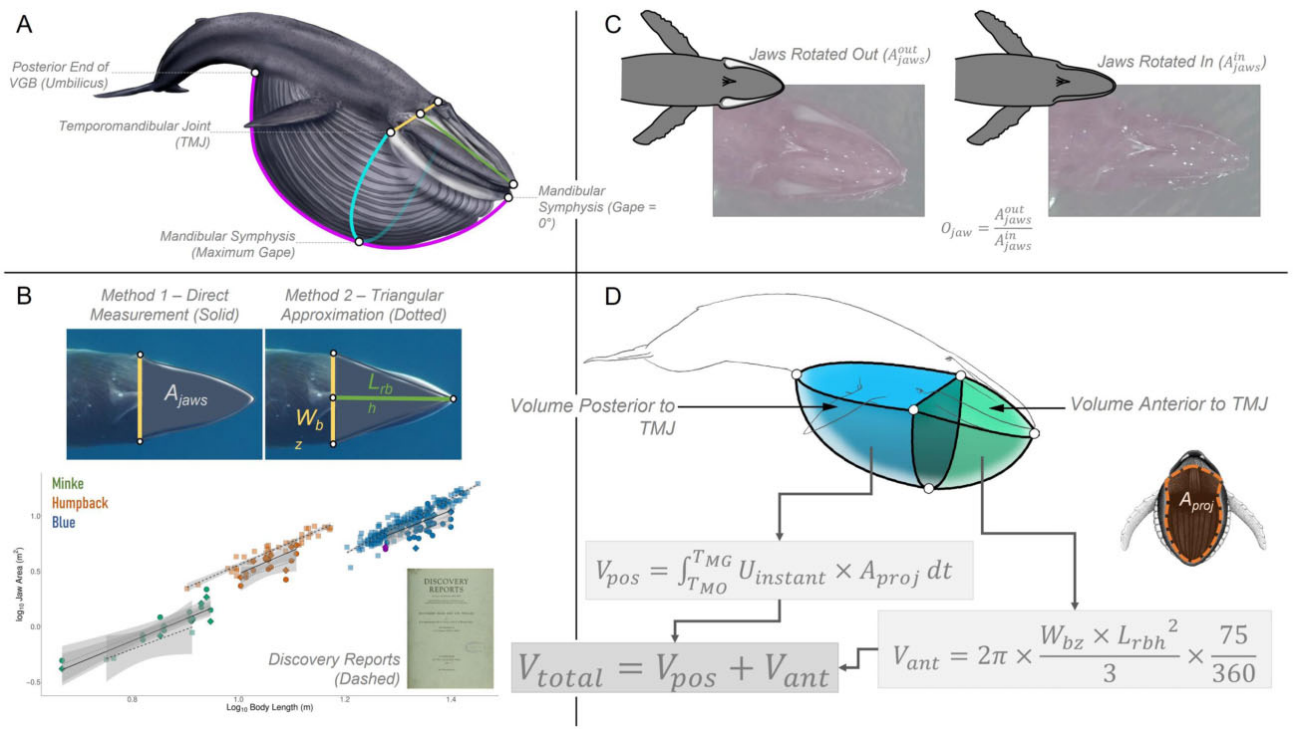
Methods used to estimate the volume of water engulfed (*M_W_*) during a lunge. The top-left quadrant (A) shows an illustration of a blue whale with the relevant morphological measurements outlined. The bottom-left quadrant (B) shows our two methods for measuring jaw area, directly and through a triangular approximation using the bizygomatic width (*W_bz_*) and the length from rostrum to blowhole (*L_rbh_*). Also shown is a comparison of *A_jaw_* against *L_body_* for our two methods (Method 1: Solid line and diamonds; Method 2: Dotted line and circles) and data from the *Discovery Reports* (dashed line and transparent squares) (Mackintosh, 1929; Mackintosh, 1942). The top-right quadrant (C) shows UAS images of a humpback whale with its jaws rotated out and rotated in, allowing us to calculate a jaw rotation factor (*O_jaw_*) and correct our jaw area measurements for jaw rotation that occurs during the lunge. The bottom-right quadrant (D) shows a schematic of the engulfed water mass modeled as two quarter-ellipsoids (blue and green) as well as the equations that we used to calculate the volume of water engulfed during a lunge.

For each animal, we measured the total length of the body (*L_body_*) as the tip of the lower jaw to the fluke notch. (A full list of symbols appears in Table 3). We measured the maximum diameter (width; *w_max_*) of each animal posterior to the flipper insertion. We measured the area between the lower jaws (*A_jaw_*) two different ways (Fig. 3(B)). First (Method 1), we used the area tool in MorphoMetriX to directly measure the entire area defined by the lower jaws from a horizontal line at the level of the bizygomatic width (*W_bz_*) to the tip of the lower jaw. Second (Method 2), we measured the bizygomatic width and the distance from the tip of the rostrum to the blowhole (*L_rbh_*) and modeled *A_jaw_* as an isosceles triangle using the equation:

(1) \begin{equation*}{A}_{jaw} = \frac{{{W}_{bz}{L}_{rbh}}}{2} \times {O}_{jaw},\end{equation*}

where *O_jaw_* is a correction factor to account for the outward rotation of the mandibles that occurs during the lunge (Lambertsen et al., 1995). Using same-altitude still images from a UAS-obtained video taken of a humpback whale swimming at the surface in southeastern Alaska, we calculated *O_jaw_* as the difference between the outwardly and inwardly rotated jaws at the surface immediately following a foraging dive (Fig. 3(C)). Combining our measurements with this *O_jaw_* estimate gave us the most accurate geometric model possible for our subsequent energetic calculations.

As a final check of our UAS-based morphological jaw measurements, we compared against similar values taken from deceased animals documented in the *Discovery Reports* (Mackintosh, 1929; Mackintosh, 1942; Kahane‐Rapport and Goldbogen, 2018). We have included these values for *A_jaw_* in Figure 3(B) and in the supplemental material for *W_bz_* and *L_rbh_* (Fig. S1) along with additional regression equations in Table S1.

### Video analysis of lunging body conformation

We compiled tag camera and UAS video sequences showing the full body (head and tail) throughout the entirety of a feeding lunge for Antarctic minke (tag *n* = 4; UAS *n* = 4), humpback (tag *n* = 75), fin (UAS *n* = 6), blue (tag *n* = 9; UAS *n* = 3), and Omura's whales (UAS *n* = 4). General information about all collected footage is included in Table S2. From these videos, we identified the moment of mouth opening, and subsequent mouth closure, and then qualitatively assessed the movement of the tail throughout the open mouth period.

### Measurement of lunging kinematics

Using a subset of deployments encompassing ≥50 measured lunges, we determined that a sample size of ∼25 randomized feeding lunges for an individual whale greatly reduced data processing time and produced stable mean kinematic values that were comparable to larger sample sizes (see supplemental information S1 and Figs. S4 and S5 for details). To ensure that we had accurate and representative values for each of our kinematic parameters, we chose a slightly higher number of randomized feeding lunges (30) for each individual whale. To assess kinematic similarity between lunges, our randomized sample was taken from the entire pool of “middle” lunges for that deployment, with “middle” lunges defined as lunges that were not the first, last, or solitary lunges from a feeding dive. To be included for subsequent analyses, the velocity profile for a lunge had to adhere to the standard pattern present in most lunges, namely accelerative during the prey-approach stage and decelerative during engulfment (Figs. 1 and 3).

For each of the randomized lunges, we measured the depth from the tag's pressure sensor as well as the forward speed of the animal at multiple times that were important for estimating the energetics of lunge feeding Fig. 1: the start of fluking after a period of gliding leading up to the lunge (*T_fluke_* and *U_fluke_*), the beginning of the steep acceleration leading up to the lunge (*T_accel_* and *U_accel_*), the position of maximum speed leading up to the lunge (*T_max_* and *U_max_*), the start of the steep deceleration that corresponds closely with mouth opening (*T_MO_* and *U_MO_*; Cade et al., 2016; Cade et al., 2020), the end of the steep deceleration period that corresponds closely with mouth closure (*T_MC_* and *U_MC_*), and the position of minimum speed that occurs within five s after the end of the steep deceleration period (*T_min_* and *U_min_*). We also calculated the duration of the acceleration period (*ΔT_accel_*) between the start of acceleration and the maximum speed, the duration of the potential adjustment period (*ΔT_adjust_*) between the maximum speed and the start of the steep deceleration, and the duration of the deceleration period (*ΔT_decel_*) between the start and end of the steep deceleration. Using the y-axis gyroscope signal, we measured the beginning (*T_gyr1_*), approximate halfway point (*T_gyr2_*), and end (*T_gyr3_*) of the final oscillation that occurs during the steep deceleration period of the lunge.

For each lunge, we determined the period of each tailbeat between *T_fluke_* and *T_gyr1_* including the final oscillation starting at *T_gyr1_* and ending at *T_gyr3_* (Fig. S2), using methods outlined by Gough et al. (2019; 2021). Next, we compared the period of the final oscillation (*ΔT_finOs_*) against *ΔT_decel_* for that same lunge.

We used allometric equations given by Kahane-Rapport and Goldbogen (2018) to calculate the length of the ventral groove blubber (VGB) (*L_VGB_*). We then compared these values against the distance traveled during each lunge (*Δ_distance_*) (Fig. S3)

### Calculating thrust and drag forces during the deceleration phase

During engulfment, neutrally buoyant rorquals are thought to sustain three types of forces during engulfment (Potvin et al., 2009; Potvin et al., 2012; Potvin et al., 2020): Propulsive thrust (*Th*) from the tail; shape drag (D_shape_), as generated by the flows externally moving about the body; and engulfment drag (D_engulf_), as generated in reaction to the forward push by the VGB musculature to accelerate the engulfed water mass up to the speed of the whale. Using the kinematics of each lunge, we were able to calculate the time-averaged engulfment drag (*D_engulf_*) experienced by that whale as follows (Potvin et al., 2020):

(2) \begin{equation*}{D}_{engul\!f} = \frac{{{M}_W{U}_{MC}}}{{\Delta {T}_{decel}}}\end{equation*}

with *M_W_* corresponding to the mass of the engulfed prey-water mixture, here estimated from UAS-derived allometric relationships reported in the literature (Kahane‐Rapport and Goldbogen, 2018).

Using simple equations of motion, we derived another relationship connecting the so-called “force residual” (*D_shape_—Th*) to the body deceleration measured by the tags. Including the slope of the deceleration allowed us to estimate the time-averaged impact of these force residuals as follows (Potvin et al., 2021):

(3) \begin{equation*}{D}_{shape} - Th = {M}_{kg}\frac{{\left( {{U}_{MO} - {U}_{MC}} \right)}}{{\Delta {T}_{decel}}} - {D}_{engul\!f}\end{equation*}

with *M_kg_* as the unladen body mass of the animal (i.e., without the engulfed prey-water mixture). All the lunges in our study follow the same general acceleration-deceleration pattern in which *U_MO_* > *U_MC_*, thereby leading to the signs shown in Eq. 4. Dividing this residual by the duration of the deceleration (*U_MO_ − U_MC__/_ΔT_decel_*) provides insight into tail propulsion generation during the deceleration phase of the lunge. A positive force residual signals that shape drag is higher than the thrust produced by the tail and is slowing the animal down more quickly than expected by engulfment drag (*D_engulf_*) alone. Alternately, a negative value denotes that thrust exceeds shape drag, causing a longer deceleration phase than expected. Figure 5 shows this relationship through density plots for each species. It should be noted that *D_engulf_* is a directly calculable quantity (Eq. 2) using our current data and methods, but *D_shape_* and *Th* must be inferred. With regards to “fluke-through” versus “acceleration-coasting” engulfment, tag measurements showing tail heaving and measurable acceleration (i.e., propulsive thrust being greater than the sum of *D_shape_* and *D_engulf_*) during the mouth-open period would characterize the former, while the absence of tail heaving coupled with measurable deceleration characterize the latter.

### Calculating lunging engulfment volume

Engulfment capacity (*V_total_*) has historically been a calculated parameter rather than measured, obtained from assumed quarter-ellipsoids modeling the filled buccal cavity (Goldbogen et al., 2010; Goldbogen et al., 2012; Kahane‐Rapport and Goldbogen, 2018). Although tag-based estimates were attempted in Cade et al. (2016), we provide a more direct field-based approach to its determination. For each lunge, we used *A_jaw_* and the forward speed during the deceleration phase to calculate the amount of prey-laden water engulfed during that lunge. We set the maximum gape to be 75° using videos of lunging whales for context. We assumed that each whale opened its mouth continuously from a closed position to maximum gape (*T_MG_*) throughout the first third of the deceleration duration (*ΔT_decel_*), remained open at maximum gape for another third, then closed its mouth from maximum gape to a closed position over the course of the final third (Goldbogen et al., 2007). The gape angle at each time-step was determined by dividing the total gape angle change (75° to open and 75° to close, leading to 150° total change in gape over the course of the deceleration phase) by the combined duration of the mouth opening and closing portions of the deceleration phase (*d_decel_*), and then multiplying by the number of time-steps that elapsed since the start of the deceleration phase. The resulting measurement was then converted from degrees to radians to give our instantaneous gape angle (*G_instant_*). With this value, we calculated the projected area of the mouth (*A_proj_*) using the equation:

(4) \begin{equation*}{A}_{proj} = {A}_{jaw}\sin \left( {{G}_{instant}} \right)\!.\end{equation*}

Multiplying *A_proj_* by the instantaneous speed of the animal (*U_instant_*) at each time-step between mouth opening and maximum gape produced a series of water volumes that could be summed (*V_pos_*):

(5) \begin{equation*}{V}_{pos} = \mathop \int \nolimits_{{T}_{MO}}^{{T}_{MG}} {U}_{instant}{A}_{proj}dt\end{equation*}

and added to an ellipsoid-based geometric model for the anterior portion of the engulfment apparatus (*V_ant_*) at a smaller gape angle of 75^o^ (vs. 90^o^) (Goldbogen et al., 2010; Goldbogen et al., 2012; Kahane‐Rapport and Goldbogen, 2018):

(6) \begin{equation*}{V}_{ant} = 2\pi \frac{{{W}_{bz}{L}_{rbh}^2}}{3} \cdot \frac{{75}}{{360}}\end{equation*}

The total volume of prey-laden water engulfed during the lunge (*V_total_*) thus follows,

(7) \begin{equation*}{V}_{total} = {V}_{pos} + {V}_{ant}\end{equation*}

an estimate which leads to the engulfed mass *M_W_* after multiplication by the density of seawater (*ρ*). These equations are laid out schematically in Figure 3(D). This model, also known as “synchronized engulfment", assumes that whales time water engulfment such that the volume posterior to the temporomandibular joint (TMJ) is full and brought up to the speed of the whale at the final moment of maximum gape (Potvin et al. 2010). If this is the case, we can model the mouth closure portion of engulfment using the geometric model detailed above. To better understand the accuracy of our water engulfment model, we compared our values of *V_pos_* and *V_ant_* against geometric equations of the same volumes taken from Kahane-Rapport and Goldbogen (2018).

### Calculating energetic cost of lunging

To determine the metabolic cost of a lunge (*E_cost_*), we used equations originally discussed in Potvin et al. (2021):

(8) \begin{equation*}{E}_{cost} = {E}_{accel} + {E}_{decel} + cetER\left( {\Delta {T}_{accel} + \Delta {T}_{decel}} \right)\end{equation*}

with the so-called “ceteral” term *CetER* accounting for the metabolic expenditure rate sustained by the organs and tissues external to the VGB musculature and locomotor apparatus. On the other hand, the terms in *E_accel_* and *E_decel_* correspond to the (metabolic) cost incurred by the tail during the acceleration phase (Fig. 1), and VGB musculature and tail during the deceleration phase and related to the corresponding mechanical work as follows:

(9) \begin{equation*}{E}_{accel} = \frac{{{W}_{flukes\left( a \right)}}}{{{\mu }_{prop}{\mu }_{met}}}\end{equation*}

(10) \begin{equation*}{E}_{decel} = \frac{{{W}_{flukes\left( d \right)}\ + \ {W}_{VGB}}}{{{\mu }_{met}}}\end{equation*}

Parameter *μ_met_* is the metabolic efficiency, herein estimated at 0.25; and *μ_prop_*, the propulsive (Froude) efficiency estimated at 0.80 (Fish and Rohr, 1999).

In the acceleration phase, the mechanical work performed by the flukes (*W_flukes_*) was calculated from the equation:

(11) \begin{eqnarray*} {W}_{flukes} &=& \frac{1}{2}{M}_{kg}\left( {{U}_{MO}^2 - {U}_{MC}^2} \right) + {W}_{parasite}\nonumber\\ && +\, \frac{1}{2}k{M}_{kg}\left( {{U}_{MO}^2 - {U}_{MC}^2} \right),\end{eqnarray*}

where *k* as an “added mass” coefficient set at 0.05 for humpbacks and 0.03 for all other species (Potvin et al., 2020; Potvin et al., 2021), *M_kg_* the mass of each animal derived from allometric equations provided in Kahane-Rapport and Goldbogen (2018). *W_parasitic_* is the parasitic drag work calculated as (Potvin et al., 2021):

(12) \begin{eqnarray*} {D}_{parasit\!ic} &=& \rho {S}_{wet}\frac{{0.072}}{{{{\left( {R{e}_{MO}} \right)}}^{0.2}}}\left[ 1 + 1.5{{\left( {\frac{{{w}_{max}}}{{{L}_{body}}}} \right)}}^{\frac{3}{2}}\right.\nonumber\\ &&\left. +\, 7.0{{\left( {\frac{{{w}_{max}}}{{{L}_{body}}}} \right)}}^3 \right]\frac{{{T}_{accel}}}{{3.8\left( {{U}_{MO} - {U}_{MC}} \right)}}\nonumber\\ &&\times \, {U}_{MO}^{0.2}\left( {{U}_{MO}^{3.8} - {U}_{MC}^{3.8}} \right)\end{eqnarray*}

with *ρ* as (again) the density of seawater, *S_wet_* the wetted surface area of the whale calculated from Gough et al. (2021), and *Re_MO_*, the Reynolds number at the speed of mouth opening.

The mechanical work performed during the deceleration phase by the musculature embedded in the VGB (*W_VGB_*) was calculated per Potvin et al. (2021), but with the shape drag term replaced by the work carried out by the force residual (*D_shaoe_—Th*):

(13) \begin{equation*}{W}_{VGB} = \frac{1}{2}{M}_{kg}{U}_{MO}^2\left( {\frac{{{U}_{MC}}}{{{U}_{MO}}}} \right)\left( {\frac{{{M}_W}}{{{M}_{kg}}}} \right)\left( {1 + \frac{{{U}_{MC}}}{{{U}_{MO}}}} \right)\end{equation*}

The work (*W_flukes(d)_*) carried out by the tail during the slow tailbeat has been omitted due its unsteady nature, which prevents the use of previous approaches to steady-state cetacean propulsion calculations (Fish and Rohr, 1999; Gough et al., 2021) (Eq. 13 is derived in supplemental information S2). Per units of body mass, such a term is expected to scale with the ratio of fluke surface area -to- body volume, in contrast to the body volume -to- body volume scaling found in the kinetic energy term implicit in Eqs. 13 and S2. Such omission is expected to be small in relation to the work by the VGB (Eq. 13) in the case of the large whales (humpback, fin, and blue whales), but possibly more significant with the minke whales. How “small” or “substantial” the omission is currently unknown.

### In Potvin et al. (2021) the ceteral term was estimated as

(14) \begin{equation*}cetER\left( {\Delta {T}_{accel} + \Delta {T}_{decel}} \right) = {f}_{Met}\left( {4.1{M}_{kg}^{0.75}} \right)\end{equation*}

with *f_Met_* as a metabolic correction factor taken from the basal metabolic rate listed in Hemmingsen, (1960). These metabolic estimates are unverified for large whales, but we have used the same scaling exponent of 0.75 taken from Kleiber (1961) to maintain consistency with previous studies. An alternate approach is to approximate this ceteral expenditure as the arithmetic average of two approximations, namely, one where the ceteral expenditures are negligible (*CetER (ΔT_accel_ + ΔT_decel_*) ∼ 0) (as hinted in Goldbogen et al. (2019a)) and one in which they are similar to those of the VGB and locomotor expenditures (*CetER (ΔT_accel_ + ΔT_decel_) ∼ E_accel_ + E_decel_*). The resulting total cost (derived in the supplemental information S3) becomes:

(15) \begin{equation*}{E}_{cost} = 1.5\left( {{E}_{accel} + {E}_{decel}} \right)\end{equation*}

### Calculating lunge energetic intake

Prey energy density and biomass estimates were taken from Goldbogen et al. (2019b) and used to calculate the prey energy contents per kg of water (*E_prey_*) in both the Antarctic and Monterey Bay environments. These prey density estimates were extrapolated from echosounder surveys of each location, so we combined our estimates for the two locations together into a single density estimate to remove any effect of location that could not be directly or accurately related to the scale of an individual lunge. We calculated the energetic gain for a lunge (*E_gain_*) with the equation:

(16) \begin{equation*}{E}_{gain} = {M}_W{E}_{prey}{\mu }_{prey},\end{equation*}

where *μ_prey_* was the digestive efficiency, estimated as 0.84 (Goldbogen et al., 2019b).

### Estimating foraging efficiency at lunge, dive, and day timescales

We determined the energetic efficiency of a given lunge as:

(17) \begin{equation*}FE = \frac{{{E}_{gain}}}{{{E}_{cost}}}\end{equation*}

For each lunge, we then calculated the duration of the encompassing dive (*d_dive_*), as well as the number of lunges that occurred during that dive (*ln_dive_*). By multiplying the *E_cost_* and *E_gain_* values for that lunge by the number of dives and, after adding in a term for excess metabolic expenditure for the non-lunging portion of the dive, we estimated the energetic ratio on the dive scale (*FE_dive_*). Finally, we performed this calculation again using a 24-h period and estimated the number of lunges per day (*ln_day_*) taken from Savoca et al. (2021) to obtain the energetic ratio on the day scale (*FE_day_*). All the symbols used throughout the manuscript are described in Table 3.

### Modeling minimum lunging speeds

Assuming constant deceleration and a decelerative force consisting mostly in engulfment drag (Potvin et al., 2020; Potvin et al., 2021), we used the kinematics of the deceleration phase of each lunge to estimate the absolute minimum speed (*U_mom_*) required to enable that animal to completely fill its buccal cavity on momentum while sustaining the total force estimated from the decelerations measured in the field:

(18) \begin{equation*}{U}_{mom} = \sqrt {2{L}_{VGB}\left( {\frac{{{U}_{MO} - {U}_{MC}}}{{\Delta {T}_{decel}}}} \right)} \end{equation*}

**Fig. 4:**
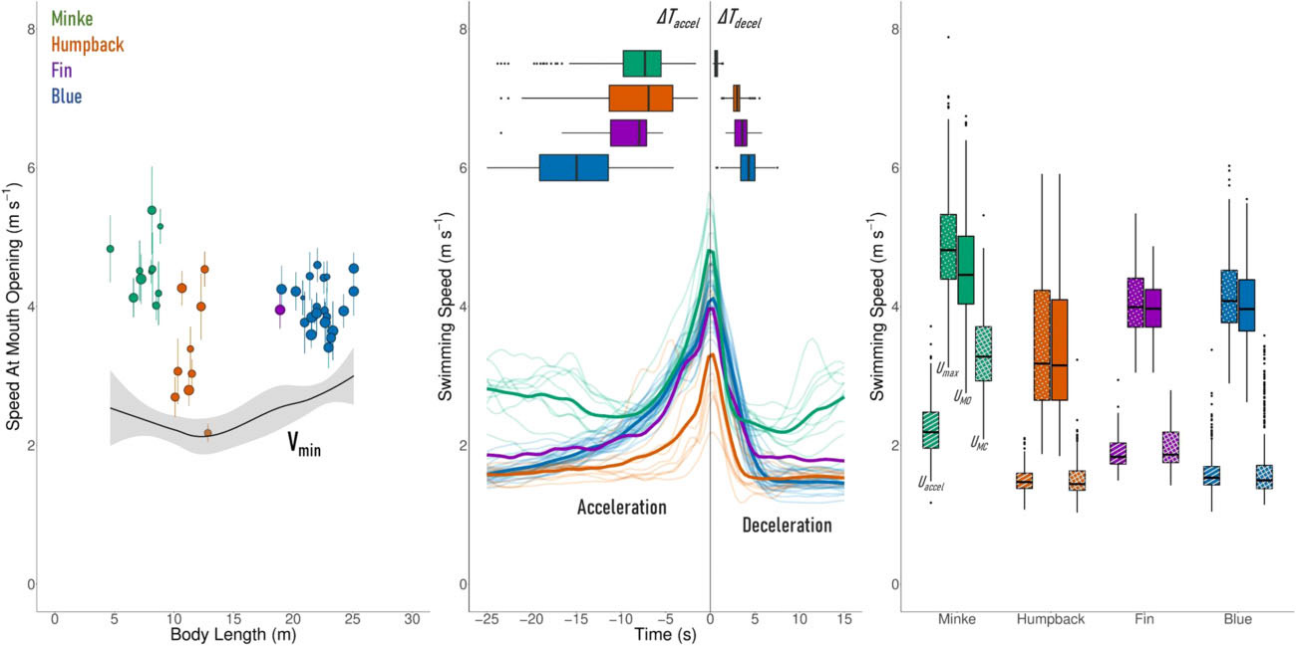
Energetically important lunge-associated swimming speeds. The first graph (A) shows the mean *U_MO_* speed for each whale in relation to body length, with vertical lines denoting the distance from the 25th to the 75th percentiles for that animal's data. This graph also displays a curve fit of *U_mom_* across our body size range. The second graph (B) shows the average speed trace for each whale (faded lines) as well as the average speed trace for each species (bold lines) starting from 25 s prior to the start of the deceleration and ending 15 s after the same position. The overlaid boxplots show the timing of the start of the acceleration phase (left of the vertical zero line) and the timing of the end of the deceleration phase. The third graph (C) shows the median, 25th and 75th percentiles, and spread of the data as boxplots for each species for the *U_accel_* (striped), *U_Max_* (dotted), *U_MO_* (no pattern), and *U_MC_* (crosshatched) speeds.

The calculated curve for *U_mom_* is shown in Figure 4A.

### Statistical testing

All statistical tests were performed in RStudio (v.1.4.1106) using the “stats” and “lme4” packages (RStudio Team, 2020). Linear relationships were coded as “ordinary-least-squares” regressions. Significance levels were set to *α* = 0.05 throughout our analyses.

## Results

### Body conformation during lunge

From tag and UAS videos of several rorqual species at a wide range of body size and geographic locations, we determined that the final full tailbeat (upstroke followed by a downstroke) is typically timed to finish just prior to mouth opening. Animals complete a final downstroke (Fig. 2(A)), then open their mouth as the tail returns to the neutral position or slowly arches to a top-of-beat position as the animal's upper jaw and head pitch upward (Fig. 2(B–D)). These movements result in an upward-pitched u-shaped body posture during the open mouth period. As the mouth begins to close, the tail slowly returns to the neutral position and the body straightens (Fig. 2(E–F)). All video sequences showed this same progression of body postures, regardless of whether the lunge occurred at the surface or at depth.

A kinematic comparison between *ΔT_finOs_* and *ΔT_decel_* showed that the final gyroscope oscillation was longer than the deceleration phase in most cases, with mean *ΔT_finOs_* values found to be 2.29s ± 0.1 for the Antarctic minke (344% longer than *ΔT_decel_*), 4.19s ± 0.28 for the humpback (43% longer than *ΔT_decel_*), 4.8s for the fin (36% longer than *ΔT_decel_*), and 7.46s ± 0.27 for the blue whale (79% longer than *ΔT_decel_*). This result suggests that the deceleration phase could occur entirely during the final gyroscope oscillation.

### Morphological measurements

Our morphological data are shown in Table 1 for each species included in our analyses. The Antarctic minke whale was the smallest species in our study, with a total length range from 4.65 m to 8.89 m, an estimated body mass range from 2358 kg to 7730 kg, and directly measured *A_jaw_* values (Method 1) ranging from 0.42 m^2^ to 1.85 m^2^. Our second biggest species was the humpback whale, with ranges for total length (10.10 m to 12.85 m), body mass (15873 kg to 27880 kg), and measured *A_jaw_* (2.79 m^2^ to 5.44 m^2^) that were slightly higher than for the Antarctic minke. Finally, the blue whale was the largest, with a total length range from 19.03 m to 25.09 m, a body mass range from 36148 kg to 96102 kg, and directly measured *A_jaw_* values ranging from 5.11 m^2^ to 12.34 m^2^. The solitary fin whale fell near the very low end of the blue whale range for all morphometric measurements.

**Table 1:** Species-specific means (± se) for important kinematic and energetic parameters

		**Morphometrics**			**Kinematics**				**Energetics**			
**Species**	**Number of individuals**	**L_body_ (m)**	**M_kg_ (kg)**	**Direct A_jaw_ (Modeled) (m^2^)**	**d_accel_ (s)**	**d_decel_ (s)**	**U_MO_ (m s^–1^)**	**V_min_ (m s^–1^)**	**M_W_ (m^3^)**	**E_cost_ (kJ)**	**E_gain_ (kJ)**	**E_ratio_ (Lunge)**
**Antarctic minke**	10	7.6 ± 0.4	5961 ± 521	1.12 ± 0.13(1.21 ± 0.14)	7.47 ± 0.61	0.67 ± 0.03	4.57 ± 0.14	2.53 ± 0.14	3.16 ± 0.51	834.92 ± 100.96	8192.70 ± 1332.42	10.89 ± 1.28
**Humpback**	9	11.4 ± 0.3	21464 ± 1427	4.02 ± 0.31(3.37 ± 0.24)	7.63 ± 0.95	2.93 ± 0.11	3.33 ± 0.26	1.99 ± 0.12	21.15 ± 1.84	1579.61 ± 338.21	54795.16 ± 4764.22	46.71 ± 7.01
**Fin**	1	18.9	39501	5.25(5.01)	8.97	3.53	3.95	2.48	39.87	3750.35	103295.8	28.64
**Blue**	22	22.3 ± 0.3	64590 ± 3154	9.10 ± 0.34(7.84 ± 0.32)	17.54 ± 0.77	4.17 ± 0.20	4.01 ± 0.07	2.75 ± 0.07	80.55 ± 4.01	6919.21 ± 350.21	208701.10 ± 10537.90	32.34 ± 1.61

For *A_jaw_*, we found that direct measurements (Method 1) were 8.04% lower than modeled values (Method 2) for the Antarctic minke, but were 19.29% higher for the humpback, 4.79% higher for the fin, and 16.07% higher for the blue whale. Our reported *A_jaw_* measurements included an *O_jaw_* offset of 5.14% to account for outward jaw rotation during the lunge. Figure 3(B) presents regressions of *A_jaw_* (methods 1 and 2 as well as data from *Discovery Reports* [Mackintosh, 1929; Mackintosh, 1942]) against body length and Table 2 presents the regression equations for each of these three methods. For the Antarctic minke, we found similar scaling exponents for all three methods (direct = 1.98; triangular = 1.90; *Discovery Reports* = 1.76), the humpback showed more variation between the triangular approximation (1.67) and the other methods (direct = 2.05; *Discovery Reports* = 2.08), and the blue whale showed the most variation between all three methods (direct = 1.88; triangular = 1.45; *Discovery Reports* = 2.49).

**Table 2:** Regression equations (log_10_ transformed) for relationships shown in Figures 3, 6, and 7. Data from *Discovery Reports* can be found in Mackintosh (1929; 1942).

**Jaw Area (m^2^) vs. Body Length (m) (** Figure 3 **B^3^)**	**Linear equation**	**R2**	**P-value**
Antarctic Minke—Direct Measurement[Triangular Approximation](Discovery Reports)	ŷ = 1.98x–1.71[ŷ = 1.90x–1.61](ŷ = 1.76x–1.61)	0.86[0.81](0.86)	<0.001[<0.001](<0.001)
Humpback—Direct Measurement[Triangular Approximation](Discovery Reports)	ŷ = 2.05x–1.57[ŷ = 1.67x–1.25](ŷ = 2.08x–1.53)	0.58[0.44](0.93)	0.003[0.017](<0.001)
Blue—Direct Measurement[Triangular Approximation](Discovery Reports)	ŷ = 1.88x–1.58[ŷ = 1.45x -1.06](ŷ = 2.49x–2.33)	0.42[0.30](0.92)	<0.001[0.012](<0.001)
**Energetic Cost (kJ) vs. Swimming Speed (m s^–1^) (** Figure 5)			
Antarctic Minke	ŷ = 2.32x + 1.35	0.49	<0.001
Humpback	ŷ = 2.37x + 1.90	0.91	<0.001
Fin	ŷ = 1.94x + 2.41	0.79	<0.001
Blue	ŷ = 2.04x + 2.60	0.69	<0.001
**Mass-Specific Energetic Cost (kJ kg^–1^) vs. Swimming Speed (m s^–1^) (** Figure 5)			
Antarctic Minke	ŷ = 2.39x + 0.55	0.83	<0.001
Humpback	ŷ = 2.32x + 0.60	0.97	<0.001
Fin	ŷ = 1.94x + 0.81	0.79	<0.001
Blue	ŷ = 2.08x + 0.77	0.82	<0.001
**Energetic Gain (kJ) vs. Swimming Speed (m s^–1^) (** Figure 5)			
Antarctic Minke	ŷ = −0.10x + 3.92	<0.001	0.44
Humpback	ŷ = 0.61x + 4.41	0.27	<0.001
Fin	ŷ = 0.50x + 4.71	0.11	0.41
Blue	ŷ = 0.38x + 5.07	0.03	<0.001
**Mass-Specific Energetic Gain (kJ kg^–1^) vs. Swimming Speed (m s^–1^) (** Figure 5)			
Antarctic Minke	ŷ = −0.03x + 3.12	<0.001	0.72
Humpback	ŷ = 0.55x + 3.12	0.36	<0.001
Fin	ŷ = 0.50x + 3.11	0.11	0.22
Blue	ŷ = 0.42x + 3.25	0.05	<0.001
**Water Engulfed (m s^3^) vs. Swimming Speed (m s^–1^) (** Figure 5)			
Antarctic Minke	ŷ = −0.10x + 0.51	<0.001	0.44
Humpback	ŷ = 0.61x + 1.00	0.27	<0.001
Fin	ŷ = 0.50x + 1.30	0.11	0.41
Blue	ŷ = 0.38x + 1.66	0.03	<0.001
**Energetic Gain/Cost Ratio vs. Swimming Speed (m s^–1^) (** Figure 5)			
Antarctic Minke	ŷ = −2.42x + 2.57	0.65	<0.001
Humpback	ŷ = −1.77x + 2.51	0.89	<0.001
Fin	ŷ = −1.44x + 2.30	0.50	<0.001
Blue	ŷ = −1.66x + 2.48	0.47	<0.001
**Water Engulfed (m s^3^) vs. Total Length (m) (** Figure 6)			
Antarctic Minke—Current Model (Geometric Model)	ŷ = 2.70x–1.91(ŷ = 3.11x–2.31)	0.81	<0.001
Humpback—Current Model (Geometric Model)	ŷ = 2.34x–1.17(ŷ = 3.25x–2.15)	0.37	<0.001
Blue—Current Model (Geometric Model)	ŷ = 2.32x–1.24(ŷ = 3.67x–3.02)	0.28	<0.001
**Energetic Cost (kJ) and Gain (kJ) vs. Total Length (m) (** Figure 6)			
Energetic Cost	ŷ = 2.07x + 1.00	0.84	< 0.001
Energetic Gain	ŷ = 2.86x + 1.48	0.93	<0.001
**Foraging Efficiency vs. Total Length (m) (** Figure 6)			
Lunge-Scale	ŷ = 0.78x + 0.48	0.30	< 0.001
Dive-Scale	ŷ = 0.63x + 0.51	0.28	<0.001
Day-Scale	ŷ = 0.29x + 0.71	0.11	<0.001

**Table 3:** Symbols used throughout this manuscript. Short descriptions are given for each symbol as a reference

**Symbol**	**Definition**
*A_jaw_*	Area of the jaw (m^2^)
*A_proj_*	Projected area of the jaws during engulfment (m^2^)
*cetER*	Energy spent by muscle and tissue external to tail and VGB (kJ)
*D_engulf_*	Engulfment drag (N)
*D_shape_*	Frictional (shape) drag (N)
*D_parasite_*	Parasitic drag (N)
*E_accel_*	Energetic cost during the acceleration phase (kJ)
*E_cost_*	Overall energetic cost of the lunge (kJ)
*E_decel_*	Energetic cost during the deceleration phase (kJ)
*E_gain_*	Energetic gain during the lunge (kJ)
*FE*	Foraging efficiency at the timescale of the lunge (dimensionless)
*FE_day_*	Foraging efficiency at the timescale of the day (dimensionless)
*FE_dive_*	Foraging efficiency at the timescale of the dive (dimensionless)
*f_Met_*	Metabolic correction factor (dimensionless)
*G_instant_*	Instantaneous mouth gape angle (degrees)
*k*	Added mass coefficient (dimensionless)
*L_body_*	Body length (m)
*L_rbh_*	Rostrum to blowhole (m)
*L_VGB_*	Length of the VGB (m)
*ln_day_*	Number of lunges that occur during a day (n)
*ln_dive_*	Number of lunges that occur during a given dive (n)
*M_kg_*	Mass of animal (kg)
*M_W_*	Mass of prey-laden water engulfed during the lunge (kg)
*O_jaw_*	Jaw outward rotation correction factor (dimensionless)
*Re_MO_*	Reynolds number at the speed of mouth opening (dimensionless)
*S_wet_*	Wetted surface area of the whale (m^2^)
*T_accel_*	The starting time of the steep acceleration prior to mouth opening (time)
*T_fluke_*	The starting time of fluking leading up to a lunge (time)
*T_gyr1_*	The starting time of the long-period gyroscope signal (time)
*T_gyr2_*	The time corresponding to the midpoint of the long-period gyroscope signal (time)
*T_gyr3_*	The ending time of the long-period gyroscope signal (time)
*Th*	Residual propulsive thrust produced during the deceleration phase (N)
*T_max_*	The time of maximum speed before mouth opening (time)
*T_MC_*	The ending time of the steep deceleration corresponding to mouth closing (time)
*T_MG_*	The time of maximum gape halfway through engulfment (time)
*T_min_*	The time of minimum speed after mouth closing (time)
*T_MO_*	The starting time of the steep deceleration corresponding to mouth opening (time)
*U_accel_*	The speed measured at *T_accel_* (m s^–1^)
*U_fluke_*	The speed measured at *T_fluke_* (m s^–1^)
*U_instant_*	Instantaneous speed of the animal during engulfment (m s^–1^)
*U_max_*	The speed measured at *T_max_* (m s^–1^)
*U_MC_*	The speed measured at *T_MC_* (m s^–1^)
*U_min_*	The speed measured at *T_min_* (m s^–1^)
*U_MO_*	The speed measured at *T_MO_* (m s^–1^)
*U_mom_*	The minimum speed necessary to lunge entirely on momentum (m s^–1^)
*V_ant_*	Volume of water anterior to the TMJ (m^3^)
*V_pos_*	Volume of water posterior to the TMJ (m^3^)
*V_total_*	Total volume of prey-laden water engulfed during the lunge (m^3^)
*W_bz_*	Bi-zygomatic width (m)
*W_flukes_*	Mechanical work performed by the flukes (kJ)
*w_max_*	Maximum diameter (m)
*W_VGB_*	Mechanical work performed by the VGB (kJ)
*Δ_distance_*	Distance travelled during the deceleration phase (m)
*ΔT_accel_*	The duration of the acceleration phase (s)
*ΔT_adjust_*	The duration of the adjustment phase (s)
*ΔT_decel_*	The duration of the deceleration phase (s)
*ΔT_dive_*	Duration of the dive encompassing a given lunge (s)
*ΔT_finOs_*	Duration of the final oscillation occurring during the mouth open period (s)
*μ_met_*	Metabolic efficiency (percentage)
*μ_prey_*	Digestive efficiency (percentage)
*μ_prop_*	Propulsive (Froude) efficiency (percentage)
*ρ*	Density of seawater (kg m^3^)

### Lunging kinematics

Some of the kinematic data are shown in Table 1 and Figure 4. The Antarctic minke consistently displayed the shortest average lunge durations (values given are the mean for all lunges performed by that individual), ranging from 6.64 s to 12.41 s, with the average acceleration phase durations ranging from 5.37 s to 10.97 s and the average deceleration phase ranging from 0.5 s to 0.85 s. We found longer average lunge durations for the humpback, ranging from 6.76 s to 16.66 s for the entire lunge period, 3.72 s to 12.88 s for the acceleration phase, and 2.41 s to 3.4 s for the deceleration phase. The fin whale displayed a longer average lunge duration (12.85 s) than the humpback, with an average acceleration phase of 8.97 s and an average deceleration phase of 3.53 s. The blue whale displayed long average lunge durations, with the entire lunge period ranging from 16.67 s to 35.47 s. This length was primarily driven by the acceleration phase, which ranged from 11.92 s to 29.43 s. The deceleration phase, by comparison, ranged from 1.59 s to 5.54 s. The average durations of the acceleration and deceleration phases are shown in Figure 4B.

We found the highest *U_MO_* speeds for the Antarctic minke whales, with the blue whales and fin whale having intermediate speeds, and the humpback displaying the slowest speeds (Fig. 4(A)). Humpback whales displayed the greatest range of average *U_MO_* speeds, (2.18 m s^–1^ to 4.54 m s^–1^; a range of 2.36 m s^–1^), with Antarctic minke whales displaying less variability (4.01 m s^–1^ to 5.39 m s^–1^; a range of 1.38 m s^–1^) and blue whales showing the least (3.41 m s^–1^ to 4.60 m s^–1^; a range of 1.19 m s^–1^). The Antarctic minke whale showed the largest differences between the *U_max_* and *U_MO_*, with the blue whale showing a smaller difference and humpback showing an extremely small difference. The *U_MC_* speeds for the Antarctic minke whales were also much higher than for the other three species. The lunge speed traces and ranges that we found for the *U_accel_, U_max_, U_MO_*, and *U_MC_* are shown in Figure 4(B) and 4(C), respectively. With the exception of the Antarctic minke, the values of *U_accel_* are similar to those of *U_MC_*, hinting at very small accelerating motions during the filtration phase. We did not find a significant relationship between *U_MO_* and lunge depth.

**Fig. 5:**
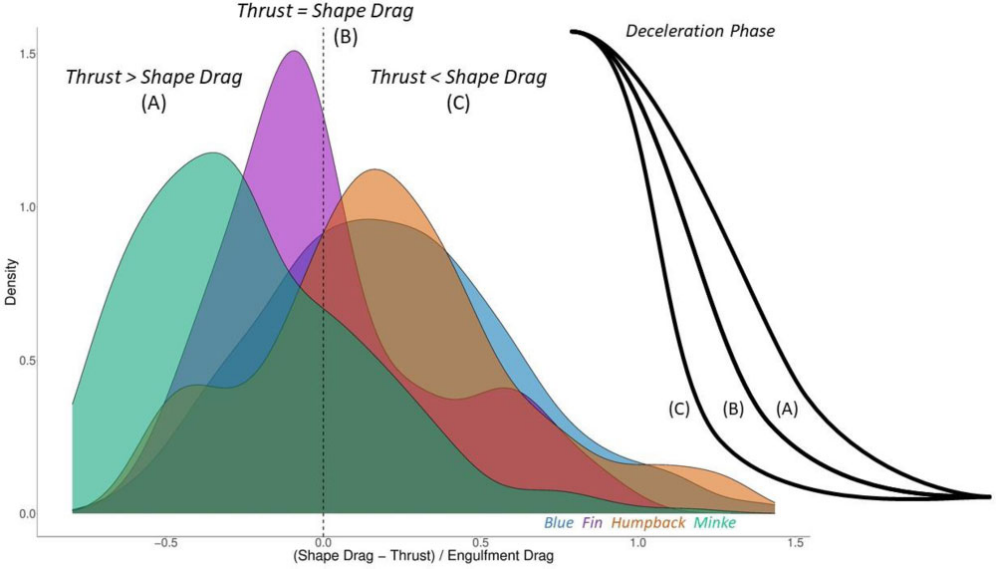
Comparison of forces during the deceleration phase of the lunge. The three conditions denote (A) when generated thrust is greater than the shape drag acting on the body, leading to a slower deceleration than predicted by engulfment drag, (B) when generated thrust is equal to shape drag, resulting in the deceleration curve predicted solely by engulfment drag, and (C) when shape drag exceeds the generated thrust, leading to a quicker deceleration than predicted by engulfment drag. The curved lines denote the deceleration phase under each condition.

The Antarctic minke whale was the only species to have a negative ratio of the normalized force residual (*D_shape_—T/D_engulf_*), with a mean value of −0.28 ± 0.08 and a range from −0.60 to 0.22 suggesting that these animals are producing excess thrust (relative to shape drag) during the lunge and a slower deceleration than predicted by engulfment drag alone. The fin whale displayed a mean value very close to zero (−0.03) and the other two species displayed positive force ratios, with the humpback having a mean value of 0.18 ± 0.11 and a range from −0.45 to 0.77, and the blue whale having a mean value of 0.19 ± 0.07 and a range from −0.41 to 0.85. These values suggest that these animals are experiencing excess shape drag and deceleration is occurring faster than predicted by engulfment drag alone. These force relationships are shown through density plots in Figure 5.

### Water engulfment model comparison

We found that our *V_pos_* model (Eq. 5) underestimated engulfment volume relative to the purely geometric model by 33.0 ± 9.3% for the Antarctic minke, 32.7% for the fin, and 25.4 ± 10.2% for the blue whale, and was similar for the humpback whale (lower by 1.3 ± 9.8%). In effect, Eq. 5 is based on the view of the buccal cavity inflating passively and compliantly, following the unkinking of the pre-folded VGB muscle fibers in the early stages of engulfment (Shadwick et al., 2013). Interestingly, these results differ from those of fluid simulations based on the engulfed slugs immediately being set into motion at the same speed as the whale by an assumed active push-forward by VGB musculature, an action that underestimated *V_pos_* by 25% relatively to the geometric model (Potvin et al., 2012).

Our *V_ant_* model, on the other hand, overestimated engulfment volume relative to the purely geometric model by 33.6 ± 5.2% for the Antarctic minke, 7.3 ± 9.1% for the humpback, and 7.8 ± 4.3% for the blue, and was similar for the fin whale (higher by 0.3%). For the full engulfment volume (*V_total_*), we found minor overestimations against the geometric model for the Antarctic minke (5.6 ± 6.5%) and humpback whales (5.3 ± 6.2%), a minor underestimation for the blue whale (8.6 ± 5.8%), and a larger underestimation for the fin whale (18.9%).

We show the mean quantity of water engulfed during a lunge (*V_total_*) in relation to body size in Figure 7(A), with mean values given in Table 1 and our regression equations given in Table 2. This value increased both within and between species and we found that the volume of water engulfed displayed negative allometry with body size for each species besides the fin whale (Antarctic minke: 2.70; humpback: 2.34; blue: 2.32), a result that differed from the purely geometric model obtained from Kahane-Rapport and Goldbogen (2018) (Antarctic minke: 3.11; humpback: 3.25; blue: 3.67). Statistical comparisons of the two models found significant differences between regression slopes for the humpback (*P* < 0.001) and blue whales (*P* < 0.001), but not for the Antarctic minke whale (*P* = 0.11).

### Lunging energetics and efficiency

**Fig. 6:**
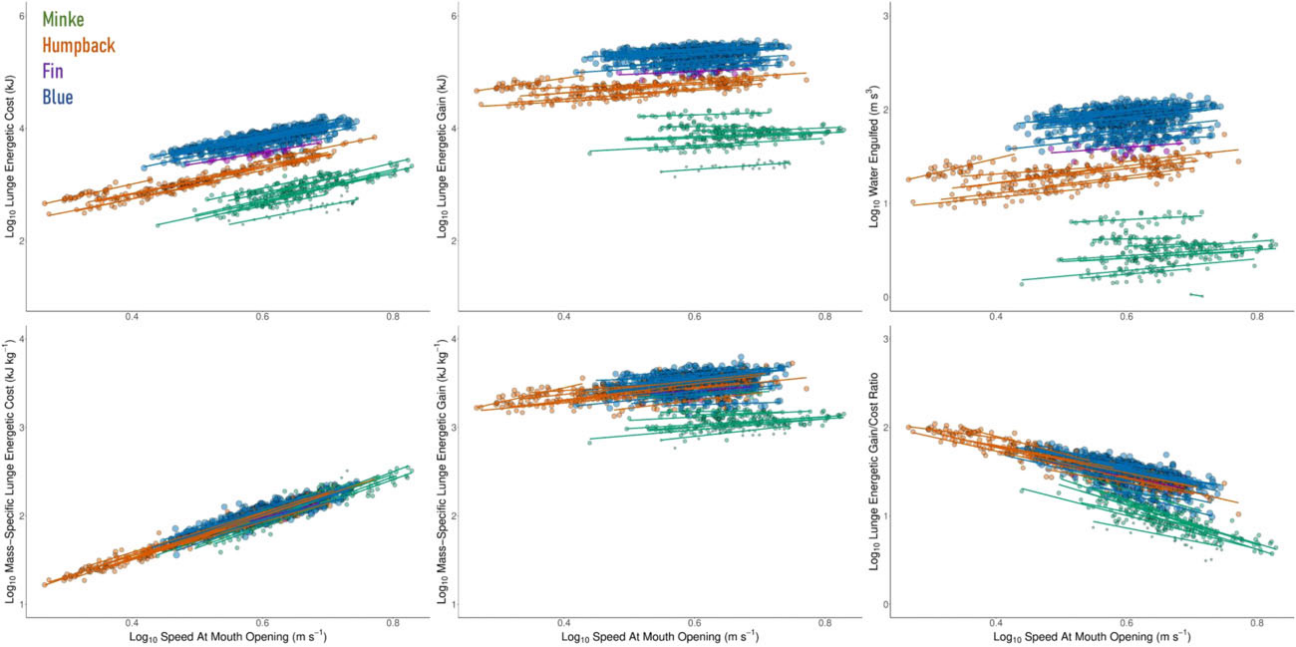
Lunge feeding energetics (*E_cost_, E_gain_*, and *FE*) as well as *V_total_* in relation to swimming speed (log_10_ transformed). *E_cost_* and *E_gain_* values are given on both an absolute and mass-specific basis. All linear regressions are displayed at the level of the individual whale.

We found that increasing *U_MO_* led to an increase in *E_cost_* but did not lead to a proportionate increase in *E_gain_*, resulting in a decrease in *FE* at higher lunging speeds. These energetic trends are shown in Figure 6 on both an absolute and mass-specific basis, as is the relationship of lunging speed with water engulfed (*V_total_*). Regression equations for each species are given in Table 2.

**Fig. 7:**
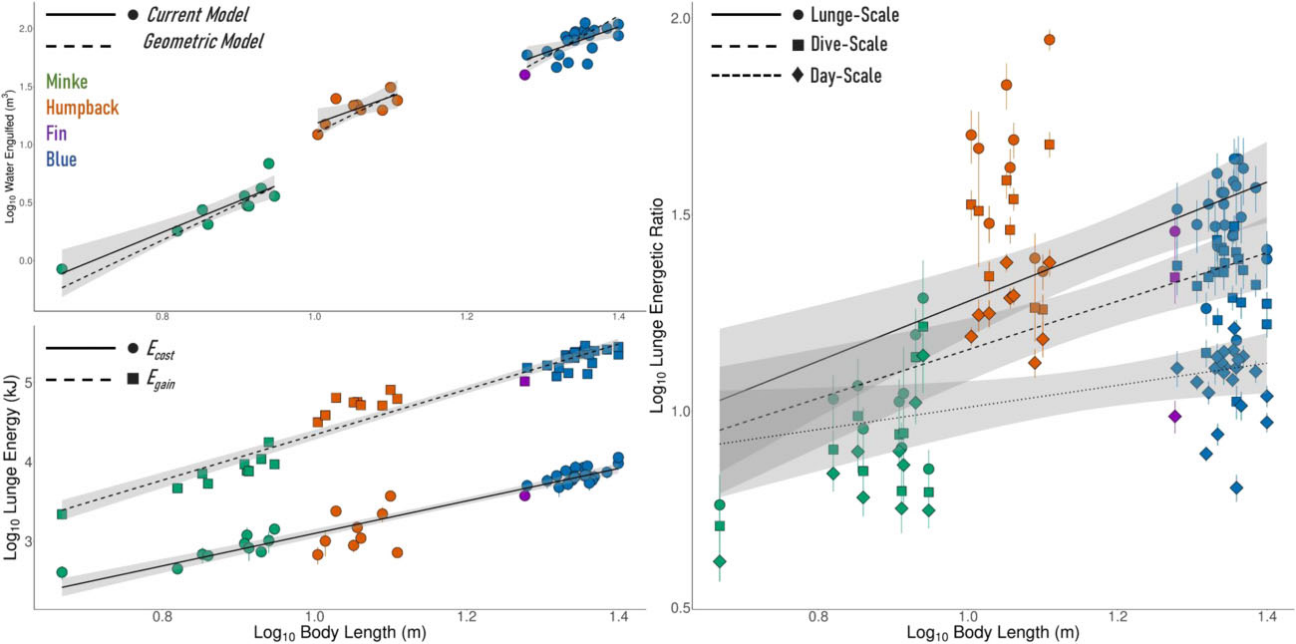
Estimates of water engulfed (*V_total_*) and lunging energetics at the lunge, dive, and day scales. The top left graph compares our estimate of *V_total_* (data points and solid lines) against an allometric estimate derived from morphological data by Kahane-Rapport and Goldbogen (2018). The bottom left graph shows the average absolute *E_cost_* (solid line and circles) and absolute *E_gain_* (dotted line and squares) for each whale. The graph on the right shows the foraging efficiency (*FE* = *E_gain_*/*E_cost_*) at the lunge scale (solid line and circles), the dive scale (*FE_dive_*; dashed line and squares), and day scale (*FE_day_*; dotted line and diamonds). Vertical lines denote the 25th and 75th percentiles of our data range.

Our estimates for lunging energetics are given in Table 1 and shown in Figure 7(B–C), with regression equations given in Table 2. Statistical comparison of the slopes for *E_gain_* and *E_cost_* showed a significant difference (*P* < 0.001). We found that the energetic efficiency (*FE*) of a single lunge increases with body length, driven primarily by increases in *E_gain_* with body length (scaling to the power of 2.86). We found that *E_cost_* also increased with body length, but to a lesser extent (scaling to the power of 2.07). Intraspecific variability away from our regression curve in both energetic parameters was greater for the Antarctic minke and humpback whales and lesser for the blue whale.

The relationship between *E_gain_* and *E_cost_* resulted in an increase in *FE* with increasing body size. This scaling relationship was less extreme on the timescale of the dive and the day. Statistical comparison of the slopes for these three slopes found significant differences between lunge-scale and dive-scale (*P* < 0.001), dive-scale and day-scale (*P* < 0.001), and lunge-scale and day-scale (*P* < 0.001).

## Discussion

Before the advent of biologging tags containing cameras and use of UAS in cetacean research, biomechanical descriptions of rorqual whale foraging behavior were based on sea surface observations (Bredin, 1985; Hoelzel et al., 1989), anatomical studies of the VGB and other tissues of the jaws and buccal cavity (Orton and Brodie, 1987; Pyenson et al., 2012; Shadwick et al., 2013), aliasing or low sample rate kinematic measurements from whale-borne tags (Goldbogen et al., 2006; Doniol-Valcroze et al., 2011), and/or hydrodynamic modeling (Cooper et al., 2008; Potvin et al., 2009, ; Potvin et al., 2010). In recent years, the combination of high sample rate tri-axial inertial sensors (Simon et al., 2012) and simultaneously recording cameras have allowed for more rigorous tests of competing hypotheses and models that describe how lunge feeding works (Cade et al., 2016; Cade et al., 2020). Most of these studies indicated that lunge filter feeding is a high cost, high benefit foraging mechanism. However, how feeding costs compare to gains among individuals and species across scale remains less understood. Our current study builds on previous work with a dataset of tagged individuals with matching UAS imagery that provides morphometric body measurements that inform hydromechanical and energetic models of engulfment (Potvin et al., 2020; Potvin et al., 2021).

### Mechanics of lunge feeding

Our video and tag data reveal a consistent sequence of body conformation changes during lunge feeding (Figs. 1 and 2). These observations suggest rorquals initiate mouth opening following several complete tailbeats during prey-approach (Fig. 1). In contrast to this series of thrust-producing tailbeats prior to mouth opening, the tag data reveal a gyroscope oscillation (defined by an oscillatory period that is relatively longer than pre-mouth opening tailbeats) that begins near the start of engulfment and continues through the end of the engulfment phase. We hypothesize that this long-period oscillation reflects body flexion adjustments that are required to balance torques and maintain trim during lunge feeding (Cooper et al., 2008). Both UAS and tag-camera footage show that the upper jaw tends to actively rise while the lower jaw drops more passively (Fig. 2). This motion could result in a lift force acting on the upper jaw, thereby causing the mouth to open wider as the anterior-dorsal body arcs away from the throat pouch. If the caudal peduncle is in a passive state during this arc, we would expect the tail to move from a downward position to a neutral position or higher, as observed in our video footage (Fig. 2).

It is unclear how much thrust is generated by the tail during this oscillation, but its long period suggests the thrust and resulting energetic cost is low or negligible compared to the overall energetic cost of the lunge. More specifically, Figure 5 suggests that a small amount of excess thrust is produced by minke whales during engulfment, that is, in amounts high enough to cancel the shape drag force (with the excess ending up as thrust per se), but in amounts insufficient to generate actual acceleration. The overall effect could be to maintain higher post-lunge speeds and allow the animal to reach the next prey patch more quickly. Larger whales, in contrast, are impacted by additional shape drag (proportional to wetted body area) which slows them down more quickly than predicted by engulfment drag. The thrust being produced by the tail appears insufficient to completely cancel out shape drag for these animals. Additionally, these animals require a longer period of time to filter out engulfed water, so maintaining a high post-lunge speed may not be as necessary, since these animals are not moving quickly to set up their next lunge (Kahane-Rapport et al., 2020). In summary, our data reveal engulfment scenarios which are neither “fluke-through” or “acceleration-coasting,” but rather a mix of the two, in which the tail is slowly beat at the onset of mouth opening to provide either full (minke) or partial (other rorquals) cancellation of shape drag. In other words, a picture of engulfment by “impulse/burst” in which this final tailbeat doesn't produce enough thrust to impart acceleration throughout engulfment, but still acts to increase or diminish the decelerative motion to modulate the precise kinematic timing of the lunge (Fig. 5).

The high lunging speeds we estimated for most tagged rorquals (Fig. 4(A)) could also necessitate a cessation of fluking before mouth opening to maintain body trim. As the mouth opens and the buccal cavity begins to inflate at high swimming speeds, the center of mass shifts and a drag force develops from the water entering the mouth (Potvin et al., 2009; Potvin et al., 2012; Potvin et al., 2020). Raising the head and adjusting the flippers and flukes might generate enough lift force to counteract this downward torque and keep the animal moving towards its prey (Cooper et al., 2008). Actively controlling the flow around the animal by fluking during engulfment might upset this balance and shift the center of mass in inefficient ways. Passive flow control from the flukes during the long-period oscillation that occurs during engulfment presumably maintains the lunge trajectory and maximizes prey capture. Active and passive flow control are both recognized mechanisms animals use to enhance hydrodynamic and locomotor performance (Fish and Lauder, 2006).

### Whale lunging speeds

Predator swimming speeds achieved during prey-capture are typically higher than non-foraging swimming speeds (Budick and O'Malley, 2000; Higham, 2007; Gough et al., 2021). High maximum speeds relative to prey escape speeds increase foraging success rates and fitness (Higham, 2007; Wilson et al., 2018). Krill exhibit maximum escape speeds below ∼0.5 m/s (Hamner, 1984; O'Brien, 1987; Letessier et al., 2013; Cade et al., 2020); therefore, if prey escape speed was the primary consideration affecting prey capture efficiency for krill-feeding rorqual whales, we would expect lower lunging speeds than observed. In addition, the physical properties of the VGB may suggest a minimum lunging speed of ∼1 m/s to fully inflate the throat pouch (Potvin et al., 2021). Instead, higher lunge speeds could generate sufficient kinematic energy needed to overcome drag and maintain momentum throughout the entire open mouth phase (Potvin et al., 2020). Our model estimations of *U_mom_* (Eq. 18) are slightly below the lunging speeds that we observed, suggesting that momentum generation could be a minimum requirement in this process for krill-feeding animals and that higher speeds may increase prey capture by limiting prey escape (Fig. 4(A)). That being said, the lunging speeds achieved by rorqual whales are only ∼2 times higher than routine swimming speeds, suggesting that these animals may be constrained by high swimming costs associated with high speeds at large body sizes.

Among the various prey types targeted by rorquals, except for slow swimming copepods targeted only by sei whales (*Balaenoptera borealis*) (Baumgartner and Fratantoni, 2008), krill have the least-developed escape responses (Hamner, 1984; O'Brien, 1987; Letessier et al., 2013; Cade et al., 2020). Cade et al. (2016) compared the lunging kinematics of humpback whales foraging on krill and fish and found that the krill-feeding animals exhibited greater stereotypy across several metrics such as maximum lunging speed and lunge duration. Fish-feeding rorquals lunge using atypical kinematics to maximize the percentage of prey caught, even if energetically costly engulfment mechanisms are used (Cade et al., 2020). In particular, the higher energetic cost of fluking during part, or all, of the open mouth portion of the lunge may be efficient if the cost is especially low, such as during low-speed lunges (Cade et al., 2020; Potvin et al., 2020), or if the energetic gain from prey intake is higher by a proportionate amount (Cade et al., 2020).

Previous studies on humpback whales, fin whales, and blue whales showed an increase in maximum lunging speed with ranked species-specific body size (Goldbogen et al., 2012; Cade et al., 2016). Our study, with UAS-derived body length measurements of tagged rorquals, suggests that mouth opening speeds do not scale with body size either within or among species (Fig. 4(A)). Some individuals of each species lunged at approximately 4 m s^–1^. However, some minke whales lunged at higher speeds (up to 6 m s^–1^), whereas some blue whales and particularly humpback whales lunged at lower speeds (down to 2 m s^–1^). It has been shown that foraging behavior can vary widely with depth, even when the prey type is the same (Friedlaender et al., 2017), but we did not find a relationship between lunge depth and lunging speeds for any of our species (Fig. 4(A)).

Our results suggest that faster lunging speeds should increase the energetic cost of the lunge, but do not lead to a commensurate increase in the energetic gain (Fig. 6), calling into question why some minke whales lunged at higher speeds than any other rorqual. Lunging at higher speeds may increase prey capture rates, as previous modeling has indicated (Cade et al., 2020); however, it is not clear that higher speeds necessarily increase krill capture, as coordinated krill escape responses are not noted in video tag deployments. The mechanical properties of the VGB in minke whales could also be different compared to other rorqual species, thus requiring higher speeds to fully inflate; however, the elastic properties of tendinous tissues may not scale with body size (Pollock and Shadwick, 1994). If minke whale prey is patchily distributed over a wide area, they may lunge at high speeds to maintain higher post-lunge speeds and allow them to move to the next prey patch more quickly.

### Impacts of scale on lunge feeding

The energetic efficiency of a rorqual foraging lunge is heavily impacted by the volume of water and prey that can be engulfed at once and the density of prey in which the whale is foraging (Friedlaender et al., 2016; Goldbogen et al., 2019b; Friedlaender et al., 2020; Kahane-Rapport et al., 2020). Allometric studies across species using data from deceased whales and geometric models assuming maximal engulfment have suggested that engulfment capacity exhibits positive allometry whereby larger rorquals can engulf relatively larger volumes of prey-laden water (Kahane‐Rapport and Goldbogen, 2018; Kahane-Rapport et al., 2020). In these models, for all species except minke whales, the largest individuals within each species (e.g., humpback whales, fin whales, and blue whales) appear capable of engulfing a volume that is greater than that of their own body. Our study sought to increase the fidelity of these volume estimations by integrating UAS-derived morphometric measurements and tag-derived kinematic profiles of the lunge, rather than a simple ellipsoid model. With this new model, we found that engulfment capacity displays negative allometry—a power exponent less than three—for minke, humpback, and blue whales. We also found wider confidence intervals that include isometry and reflect the increased variability in our dataset (Fig. 7(A)). Small changes to *A_jaw_* led to very different estimations of *V_total_* and could explain some of this variability, especially with our reduced sample size relative to previous allometric studies. As a check that this reduced sample size was not directly affecting the allometry of engulfment, we recreated the geometric model from Kahane-Rapport and Goldbogen (2018) for each species in our dataset and found isometry or slight positive allometry (Kahane‐Rapport and Goldbogen, 2018; Kahane-Rapport et al., 2020). This comparison suggests that the ellipsoid model (Goldbogen et al., 2010) assumes maximal filling and may represent a useful maximal engulfment scenario, and the inclusion of our fine-scale kinematics or morphometrics may capture less-than-optimal engulfment scenarios. However, it has not been clearly shown whether krill-feeding rorquals modulate engulfment capacity on a lunge-to-lunge basis, as suggested for fish-feeding species like humpback whales (Cade et al., 2016; Cade et al., 2020). Modulation of engulfment capacity may affect prey ingestion estimates used to determine the impact of whales on their environment (Savoca et al., 2021).

As rorqual whales increase in body size, more time is required to generate momentum and increase engulfment volume (Fig. 4(B)). For the other species in our dataset, mouth closure occurred near the measured minimum speed values, corresponding to a traveled distance proportional to the length of the VGB (Fig. S3). For minke whales, speed loss continued after the end of the steep deceleration period, with mouth closure occurring approximately halfway through the overall deceleration trend and maintaining the same distance traveled as the other species. Coupled with longer acceleration phases, minke whales were taking longer to build momentum and losing it quicker than other species (Fig. 4(B)).

If krill-feeding rorqual whales are lunging primarily on momentum without actively fluking, we can split the energetic cost and energetic gain into separate components that exist within the acceleration and deceleration phases, respectively (Potvin et al., 2021). For our calculation of energetic cost, we included an estimation of metabolic energy usage throughout the lunge based on a metabolic scaling exponent of 0.75 (Hemmingsen, 1960; Kleiber, 1961) that has been used in previous studies of cetacean energetics (Czapanskiy et al., 2021; Potvin et al., 2021). Small changes in metabolic rate can lead to high variation in the estimation of energetic cost, but without having direct measurements of metabolic rate, we used a common scaling exponent that will make comparison easier between our current study and both past and future analyses of rorqual whale energetics.

Our estimates for the energetic cost and gain of a lunge are consistent with previous studies (Goldbogen et al., 2019b; Cade et al., 2020; Potvin et al., 2021), with the cost increasing more slowly and gain increasing more rapidly with increasing body size (Fig. 7). This result suggests that many other factors could play an important role in influencing energetics including environmental variation in prey fields and variations in foraging behavior when encountering different types of prey distributions and densities.

As compared to previous work (Goldbogen et al., 2019b), we examined foraging energetics on dive- and day-scales to supplement the discrete lunge-scale and estimate energetic balance over broader timescales. We found that as timescales increased, the scaling exponent for our foraging efficiency as a function of body size decreased. This result aligns with the general trend found across taxa of decreased feeding rates at larger body sizes (Rall et al., 2012; Hoey and Bonaldo, 2018). In rorquals, this change in the foraging efficiency could result from larger whales spending a greater proportion of their dive time filtering the water engulfed during each lunge (Kahane-Rapport et al., 2020) or from greater search times in between feeding events. This additional filter or search time accrues metabolic costs without contributing directly to energetic gain, resulting in a slightly lowered energetic efficiency for larger whales at longer timescales, even if their overall energetic balance is still higher than for smaller rorquals.

## Conclusions and caveats

Due to their large size and pre-whaling abundance, rorqual whales have been shown to play an instrumental role within their environment as drivers of the nutrient cycle (Savoca et al., 2021). Estimating the magnitude of these ecosystem services requires accurate measurement of energetic intake and foraging efficiency. Through the combined use of biologging tags, UAS, and hydrodynamic modeling, our study provides greater detail than ever before on the scaling of rorqual lunge feeding kinematics and energetics. This feeding strategy has previously been described through a strict dichotomy between powered and unpowered engulfment of prey. In contrast, our analyses are the first to suggest a softer gradient, with small quantities of thrust acting to minimize the effects of drag and modulate the precise timing of engulfment. We also found that the speeds achieved during lunge feeding are higher than both known krill escape speeds and mechanical VGB inflation speeds and do not scale predictably with body size for a broader set of species than has ever been tested before. Instead, the variation in lunge speed may reflect fine-scale variation in prey that is not currently measurable on a lunge-to-lunge basis. Regardless of this variability in lunging speeds within and among species, we found that the energetic ratio of the lunge increases with body size across multiple timescales, thereby highlighting the general advantage of large body size for engulfment filter feeders. At the scale of a year, high foraging efficiency might contribute to the long migrations undertaken by many large rorqual whale species (Watanabe et al., 2015).

Moving forward, our model of engulfment could be improved in several ways pertaining to the mechanics of engulfment, the dynamics of the prey, or both. For example, our study is the first to assume that rorqual whales are not filling their buccal cavity to maximum engulfment on each lunge, but we still assume a constant maximum gape angle and standardized timing of mouth opening and closing, variables that may be highly modular between lunges. We also have little information on the precise mechanics of the VGB and how extensibility of muscle fibers may affect the speed necessary for each species to inflate their buccal cavity. In respect to the prey, we have assumed a single consistent prey density and energetic content for krill, but we know from previous work on rorquals that these variables can vary widely and impact both the mechanics and energetics of foraging, even within stereotyped krill-feeding lunges (Hazen et al., 2015; Cade et al., 2016; Guilpin et al., 2019; Goldbogen et al., 2019b; Savoca et al., 2021; Cade et al., 2021a). Recent work by Cade et al. (2020) has also shown how prey escape responses dictate the kinematics of the lunge, but similar analyses have not been performed for krill-feeding animals and their prey. Addressing these factors in future work will lead to a greater understanding of rorqual lunge feeding and how this unique foraging strategy relates to large body size.

## Data Availability

All data is available upon request. Code used to develop figures is available at https://github.com/wgough/LungeKinematics
